# Involvement of transposable elements in Alzheimer’s disease pathogenesis

**DOI:** 10.18699/vjgb-24-27

**Published:** 2024-04

**Authors:** R.N. Mustafin, E.K. Khusnutdinova

**Affiliations:** Bashkir State Medical University, Ufa, Russia; Bashkir State Medical University, Ufa, Russia Institute of Biochemistry and Genetics – Subdivision of the Ufa Federal Research Centre of the Russian Academy of Sciences, Ufa, Russia

**Keywords:** Alzheimer’s disease, carcinogenesis, miRNA, aging, transposons, retroelements, болезнь Альцгеймера, канцерогенез, микроРНК, старение, транспозоны, ретроэлементы

## Abstract

Alzheimer’s disease affects an average of 5 % of the population with a significant increase in prevalence with age, suggesting that the same mechanisms that underlie aging may influence this pathology. Investigation of these mechanisms is promising for effective methods of treatment and prevention of the disease. Possible participants in these mechanisms are transposons, which serve as drivers of epigenetic regulation, since they form species-specific distributions of non-coding RNA genes in genomes in evolution. Study of miRNA involvement in Alzheimer’s disease pathogenesis is relevant, since the associations of protein-coding genes (APOE4, ABCA7, BIN1, CLU, CR1, PICALM, TREM2) with the disease revealed as a result of GWAS make it difficult to explain its complex pathogenesis. Specific expression changes of many genes were found in different brain parts of Alzheimer’s patients, which may be due to global regulatory changes under the influence of transposons. Experimental and clinical studies have shown pathological activation of retroelements in Alzheimer’s disease. Our analysis of scientific literature in accordance with MDTE DB revealed 28 miRNAs derived from transposons (17 from LINE, 5 from SINE, 4 from HERV, 2 from DNA transposons), the expression of which specifically changes in this disease (decreases in 17 and increases in 11 microRNA). Expression of 13 out of 28 miRNAs (miR-151a, -192, -211, -28, -31, -320c, -335, -340, -378a, -511, -576, -708, -885) also changes with aging and cancer development, which indicates the presence of possible common pathogenetic mechanisms. Most of these miRNAs originated from LINE retroelements, the pathological activation of which is associated with aging, carcinogenesis, and Alzheimer’s disease, which supports the hypothesis that these three processes are based on the primary dysregulation of transposons that serve as drivers of epigenetic regulation of gene expression in ontogeny

## Abstract (ru)

Болезнь Альцгеймера поражает в среднем 5 % населения со значительным увеличением распространенности с возрастом, что свидетельствует о возможном влиянии на данную патологию тех же механизмов, которые лежат в основе старения человека. Исследование этих механизмов перспективно для разработки эффективных методов лечения и профилактики заболевания. Возможными участниками этих механизмов являются транспозоны, которые служат драйверами эпигенетической регуляции, поскольку формируют в эволюции видоспецифические распределения генов некодирующих РНК в геноме человека. Изучение роли микроРНК в развитии болезни Альцгеймера актуально, поскольку по результатам проведенных GWAS ассоциаций белок-кодирующих генов (APOE4, ABCA7, BIN1, CLU, CR1, PICALM, TREM2) трудно объяснить сложный патогенез заболевания. Кроме того, в различных долях головного мозга при болезни Альцгеймера были обнаружены специфические изменения экспрессии множества генов, что может быть обусловлено глобальными регуляторными изменениями под влиянием транспозонов. Действительно, экспериментальные и клинические исследования показали патологическую активацию ретроэлементов при болезни Альцгеймера. Проведенный нами анализ научной литературы в соответствии с базой данных MDTE DB (microRNAs derived from transposable elements) позволил выявить 28 различных микроРНК, происходящих от мобильных элементов (17 – от LINE, 5 – от SINE, 4 – от HERV, 2 – от ДНК-транспозонов), экспрессия которых специфически изменяется при данном заболевании (снижается у 17 и повышается у 11 микроРНК). Экспрессия 13 из 28 микроРНК (miR-151a, -192, -211, -28, -31, -320c, -335, -340, -378a, -511, -576, -708, -885) меняется также при старении и развитии злокачественных новообразований, что подтверждает возможное наличие общих патогенетических механизмов. Большинство из этих микроРНК произошли от LINE-ретроэлементов, патологическая активация которых ассоциирована со старением, канцерогенезом и болезнью Альцгеймера, что свидетельствует в пользу гипотезы о том, что в основе этих трех процессов лежит первичная дисрегуляция транспозонов, которые служат драйверами эпигенетической регуляции экспрессии генов в онтогенезе

## Introduction

Alzheimer’s disease (AD) is the most common neurodegenerative disease. Disease pathogenesis is caused by extracellular deposition of beta-amyloid plaques and intracellular accumulation of tau protein tangles with cell death in the brain (Barak et al., 2013). AD is detected in 62 % of patients with dementia (Swarbrick et al., 2019). In 2017, a meta-analysis revealed a 5 % prevalence of AD in Europe (3.31 % in men and 7.13 % in women) and an increase in these rates with age (7.66 % in 75–84-year-olds, 22.53 % in 85-year-olds and older). In Japan, AD occurs in 7 % of people over 65 years of age, in the USA – in 9.51 % of people over 70 years of age. In Chinese residents, the incidence of AD is 1.27 % in people 65–69 years old and 18.54 % in people 85–89 years old (Niu et al., 2017). Twin studies showed the heritability of AD to be 58 %, regardless of gender (Gatz et al., 2006).

In 2018, a genome-wide association study (GWAS) of DNA samples from 314,278 patients showed an association of the ACE, ADAM10, BCKDK/KAT8, TOMM40, VKORC1 genes with AD (Marioni et al., 2018). In 2019, a meta-analysis of GWAS results (53,042 AD patients and 355,900 healthy controls) identified 37 specific loci associated with AD in the human genome. Among them, the APH1B, BIN1, CASS4, CCDC6, NCK2, PILRA, PTK2B, SPRED2, TSPAN14 genes showed the greatest significance. However, it is difficult to explain the role of allelic variants of these genes in the pathogenesis of AD of these genes

Possible mechanisms of other AD-associated genes are shown for LILRB2 (encodes a receptor that recognizes multiple HLA alleles and may be involved in the growth of betaamyloid fibrils), ABCA1 (involved in the transfer of phospholipids to apolipoproteins), AGRN (involved in the formation of synapses of mature hippocampal neurons) (Schwartzentruber et al., 2021). In 2021, a meta-analysis showed an association of 23 different SNPs with AD, among which the highest significance was determined for rs3865444 (in the CD33 transmembrane receptor gene), rs7561528 (in the nucleocytoplasmic adapter protein gene (BIN1)) and rs1801133 (in the methylenetetrahydrofolate reductase gene (MTHFR)) (GNS et al., 2021).

In GWASs, a significant association with AD was also shown for the CLU (APOJ, encodes apolipoprotein J), CR1 (encodes complement component 3b/4b) (Lambert et al., 2009), APOE (encodes apolipoprotein E), PICALM (encodes protein phosphatidlinositol-binding clathrin assembly) (Harold et al., 2009; Ando et al., 2022), BIN1 (Ando et al., 2022) genes. Meta-analyses of GWAS results with Alzheimer’s disease showed a significant association of allelic variants of the TREM2 (encodes the trigger receptor expressed on protein 2 myeloid cells) (Guerreiro et al., 2013) and ABCA7 (Ma et al., 2018) genes.

According to numerous genome-wide association metaanalyses and large-scale genome-wide association studies, the strongest genetic risk factor for sporadic AD is the APOE ε4 allele, while the most powerful protective genetic factor is the APOE ε2 allele. This is due to the effects of APOE on β-amyloid peptide aggregation and clearance, neurofibrillary tau degeneration, microglial and astrocyte responses, and the blood-brain barrier (Serrano-Pozo et al., 2021). The production and breakdown of amyloid are also directly influenced by the AβPP, PSEN1 and PSEN2 genes, the allelic variants of which contribute to increased toxic amyloid types aggregation (Robinson et al., 2017).

The key role of genetic factors in the development of AD is evidenced by the presence of monogenic hereditary forms of the disease with an autosomal dominant type of inheritance. These forms of the disease are caused by germline mutations in the APP (amyloid precursor protein) (Rogaev et al., 1994; Goate et al., 2006), PSEN1 (presenilin-1) (Sherrington et al., 1995), PSEN2 (presenilin-2) (Levy-Lahad et al., 1995) genes. Genomic instability is important in the pathogenesis of AD, as evidenced by the pronounced association of AD with age (which is characterized by genomic instability) (Hou et al., 2017). A genomic instability component in AD may be expression changes of long non-coding RNAs, such as XIST (X-inactive specific transcript), which is considered as a potential target for AD therapy (Chanda, Mukhopadhyay, 2020).

In addition to the association of allelic variants of specific genes from DNA samples of peripheral blood leukocytes of patients with AD, a number of studies have analyzed the expression of specific genes in the brain cells of patients. The data obtained could better explain the possible mechanisms of AD pathogenesis. In 2022, a meta-analysis identified 1915 differentially expressed genes in the entorhinal cortex (the hippocampus-related part of the temporal lobe) in AD patients compared to healthy controls (Fagone et al., 2022). Earlier, in 2019, a meta-analysis of the transcriptome in AD had showed differential expression of a large number of genes in different lobes of the brain: in the temporal lobe – 323, in the frontal – 435, parietal – 1023, cerebellar – 828 genes (Patel et al., 2019). This indicates a pronounced deregulation of gene expression in the brain in AD on a genome-wide scale, a possible cause of which is the pathological activation of transposable elements (TEs), which occupy 45 % of the human genome. TEs have a global regulatory effect on the expression of all genes (as drivers of epigenetic regulation (Mustafin, Khusnutdinova, 2017)) and binding sites for transcription factors (Mustafin, 2019)

The cause of genomic instability in neurons in AD may be somatic recombinations between TEs, such as Alus and LINE1s (Pascarella et al., 2022). This is evidenced by recent results obtained from fluorescent in situ hybridization (FISH) in individual neurons of AD patients’ brain (Yurov et al., 2023), as well as experimental studies on mice with knockdown of one allele of the BMI1 gene (encodes a protein of the Polycomb group and regulates compaction of heterochromatin). In adults, BMI1 is normally expressed ubiquitously in brain neurons, but is reduced in AD. Bmi1+/– mice are characterized by neurodegenerative changes similar to AD. In this case, the loss of heterochromatin is determined mainly in the regions with repeating sequences, which include TEs or originated from them in evolution (El Hajjar et al., 2019).

TEs carry out epigenetic regulation due to their interaction with microRNAs, which evolved from TEs by various mechanisms (see the Figure), as well as by processing their transcripts to form microRNAs (Wei et al., 2016). MicroRNAs have a post-transcriptional regulatory effect on gene expression (Barak et al., 2013) and are guides for binding to DNA methyltransferases (RdDM, RNA-directed DNA methylation) with specific genomic loci, regulating expression at the transcription level (Watcharanurak, Mutirangura, 2022). Therefore, it can be assumed that the observed hypermethylation of 236 specific CpG location loci in the cerebral cortex of AD patients occurs under the influence of microRNAs upon activation of TEs (Smith et al., 2021).

**Fig. 1.**
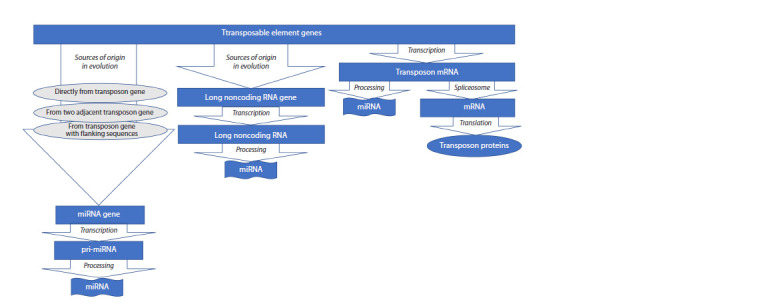
Mechanisms of origin of microRNAs from transposable elements.

TEs are divided into classes of DNA transposons (moving by a cut-and-paste mechanism) and retroelements (REs). Transposition of REs occurs by “copy-and-paste” with an intermediate RNA, from which cDNA is formed by reverse transcription. Based on the presence of long terminal repeats (LTRs), REs are classified into LTR-containing REs and non-LTR-REs. The latter include autonomous LINEs (long interspersed elements) and non-autonomous SINEs (short interspersed elements) and SVEs (SINE-VNTR-Alus). LTRREs are endogenous retroviruses (ERVs), occupying 8 % of the human genome, while LINEs (L1 and L2) occupy 21 % (Ravel-Godreuil et al., 2021).

## Role of transposable elements in Alzheimer’s disease development

The activity of TEs is under the control of epigenetic modifiers (DNA and histone methylation), as well as specific molecules, such as PRC2 (Polycomb repressive complex 2, which forms the H3K27me3 mark), DNMT1 (promotes the formation of H4K20me3), KAP1 protein (Kruppel-associated box associated protein 1, promotes the formation of H3K9me3 marks), sirtuin 6 (SIRT6, causes repression of L1 through ri-bosylation of KAP1, facilitating the interaction of KAP1 with its partners and the formation of heterochromatin in the L1 promoter region) (Ravel-Godreuil et al., 2021). According to recent data, TEs themselves are drivers of epigenetic regulation of genes through the formation of long non-coding RNAs and microRNAs from their transcripts (Mustafin, Khusnutdinova, 2017). That is, TEs are under the control of regulatory mechanisms that they drive, which indicates the presence of evolutionarily programmed self-control. Failure in this system is one of the factors of human aging (Wood, Helfand, 2013; Van Meter et al., 2014).

It is possible that the relationship between TEs and tau proteins reflects the system of mutual regulation of TEs and genes in the human genome. Indeed, inhibition of the BMI1 gene (component of the Polycomb repressive complex 1, which promotes chromatin compaction and gene silencing through E3-mono-ubiquitin ligation activity mediated by Ring1a/b on histone H2A at lysine 19 (H2Aub)) expression was found in the brain of AD patients. BMI1 gene knockout in postmitotic human neurons resulted in beta-amyloid deposition and accumulation of tau protein (because BMI1 suppresses tau protein transcription) (Flamier et al., 2018).

Modeling AD in mice by knocking out one allele of the Bmi1 gene showed the development of neurodegeneration due to derepression of TEs (El Hajjar et al., 2019). Experiments in mice show enhanced processing of non-coding RNAs from SINE B2 transcripts in the hippocampus under the influence of amyloid deposition (Cheng et al., 2020). Transcriptomic analysis showed activation of TEs (mainly ERVs) induced by aging and tau in mouse brain. Transgenic mice expressing tau protein in the brain showed an increased number of TEs’ DNA copies (Ramirez et al., 2022). G-quadruplex derived from evolutionarily conserved L1 suppresses gene expression in AD neurons (Hanna et al., 2021).

In 2018, an analysis of postmortem brain tissue samples showed that in tauopathies, decondensation of heterochromatin and decreased levels of piwi and piRNA cause deregulation of TEs. A significant increase in HERVs transcripts was also found in AD brains (Sun et al., 2018). In the same year, a study of postmortem brain tissue from AD patients (636 people) and a Drosophila model of the disease showed differential expression of several specific REs in association with the load of neurofibrillary tau tangles. In this case, global transcriptional activation of LINE1s and ERVs occurred. Tau protein-associated chromatin marks were detected at HERVFc1 location loci. Profiling of TEs in Drosophila throughout the brain showed heterogeneous response profiles, including those depending on age and genotype, activation of TEs under the influence of tau proteins (Guo C. et al., 2018).

Further studies of post-mortem brain tissue from patients with AD (60 individuals) confirmed the data on the activation of specific TEs (L1s and Alus) in AD compared with controls (Grundman et al., 2021). Analysis of blood samples from 25 late-onset AD patients revealed a significant increase in the expression of 1790 RE transcripts (LINE, LTR, SVA) before clinical phenoconversion (from normal cognitive indicators to the manifestation of AD), which the authors called a retrotransposon storm (Macciardi et al., 2022). It is possible that the data obtained by the researchers indicate the effect of a feedback relationship between TEs activated during aging and the influence of the resulting tau proteins on them, which triggers the cascade mechanism of the TEs > tau proteins > TEs relationship

Activation of REs in AD depends on the transmission of redox signals (such as complex I of the mitochondrial respiratory chain) from mitochondria to the nucleus. It is believed that this phenomenon is a side effect of general signaling from mitochondria to the nucleus, aimed at facilitating the transcription of mitochondrial genes to restore mitochondrial function (Baeken et al., 2020). As a result, DNA hypomethylation and increased expression of REs, such as LINE1s, occur (Protasova et al., 2021). In this case, a vicious circle may develop when activated REs aggravate mitochondrial pathology due to insertions into genes involved in their functioning. Thus, frequent primate-specific retrotranspositions of Alu elements into the introns of the TOMM40 gene, encoding the β-barrel protein necessary for mitochondrial transport of preproteins and associated with AD, were identified (Larsen et al., 2017).

## Association of microRNAs derived from transposable elements with Alzheimer’s disease

In 2016, G. Wei et al. created a database of microRNAs derived from TEs (MDTE DB: a database for microRNAs derived from Transposable element) (Wei et al., 2016). Due to the presence of data on the role of dysregulation of TEs in AD (Guo C. et al., 2018; Sun et al., 2018; Grudman et al., 2021; Macciardi et al., 2022), analysis of specific microRNAs presented in the MDTE DB may reveal one of the mechanisms of AD pathogenesis upon activation of TEs. In 2019, S. Swarbrick et al. conducted a systematic review of the accumulated data in the scientific literature on microRNAs associated with Alzheimer’s disease. A significant role was identified for 44 microRNAs in blood plasma, 250 microRNAs in the brain, 153 microRNAs in cerebrospinal fluid (Swarbrick et al., 2019).

Our analysis of the scientific literature allowed us to determine the association of a number of microRNAs derived from TEs that are associated with AD. In 2014, a study of the brains of rabbits modeled for AD found decreased expression of miR-576-3p (Liu et al., 2014), which was derived from L1 (Wei et al., 2016). In 2022, a reduced level of miR-576-3p was detected in the serum of people with AD (Xu et al., 2022). In 2014, a GWAS of blood samples from 158 AD patients and 155 healthy controls showed a significant difference in the expression of miR-885-5p (derived from SINE/MIR (Wei et al., 2016)) in AD (Tan et al., 2014). Further studies showed that overexpression of miR-885-5p attenuates beta-amyloidinduced neuronal damage by suppressing KREMEN1 synthesis (Pan et al., 2022).

In 2015, a comparative analysis of microRNA levels in blood samples of 48 patients with AD and 22 controls showed an increase in the expression of miR-151a (Satoh et al., 2015), derived from L2 (Wei et al., 2016), miR-3200 (Satoh et al., 2015), derived from ERVL (Wei et al., 2016), and a decrease in the expression of miR-502 (derived from L2 (Wei et al., 2016)) (Satoh et al., 2015). In the same year, a study of 127 AD patients and 123 controls revealed a decrease in the level of miR-31 in AD (Dong H. et al., 2015), which originated from L2 (Wei et al., 2016). Experiments on AD mouse models showed significant improvement in neurological parameters with lentiviral-mediated expression of miR-31 due to a decrease of beta-amyloid in the hippocampus (Barros-Viegas et al., 2020).

In 2016, an experiment in mice modeled for AD demonstrated the role of miR-211 (derived from L2 (Wei et al., 2016)), affecting NUAK1, causing the accumulation of betaamyloid and reducing neuronal survival (Fan et al., 2016). Elevated levels of miR-211 were found in another study in AD mouse models and beta-amyloid accumulation (Sierksma et al., 2018). A decrease in the expression of miR-511 (derived from L1 (Wei et al., 2016)) was found in AD, resulting in increased synthesis of the FKBP5 protein (Zheng et al., 2016). Treatment of AD mouse models with cauterization at acupuncture points of the control vessel contributed to the improvement of cognitive functions by increasing the expression of miR-511-3p (Jia et al., 2022).

In 2017, mouse models of AD showed increased levels of miR-28-3p (the miR-28 family is derived from L2 (Wei et al., 2016)) in the cerebrospinal fluid (Hong et al., 2017). In the blood serum of people with asthma, an increased concentration of miR-28-3p was also determined, compared to healthy controls. The level of this microRNA decreased with effective donepizil therapy (Zhao et al., 2020).

ERVL-derived miR-1246 (Wei et al., 2016) has been proposed as a biomarker of AD to determine its level in the blood serum of patients (Guo R. et al., 2017). A decreased miR- 545-3p level was determined in the blood plasma of patients with AD compared to controls (Cosin-Tomas et al., 2017). The miR-545 family originated from L2 (Wei et al., 2016). In AD, reduced expression of miR-325 (derived from L2 (Wei et al., 2016)) is determined, which has a post-transcriptional regulatory effect on tomosyn synthesis (impairs synaptic transmission in the brain) in the hippocampus (Barak et al., 2013). The pro-inflammatory microRNA miR-326, derived from the DNA transposon hAT-Tip100 (Wei et al., 2016), was characterized by increased expression in AD (Cai et al., 2017). Low levels of miR-342-5p (derived from SINE (Wei et al., 2016)) were detected in the worst course of AD (Dakterzada et al., 2021). SINE-derived miR-3646 was overexpressed in AD patients (Lu et al., 2021).

In 2018, increased expression of miR-320c (derived from L1 (Wei et al., 2016)) was determined in patients with AD compared to patients with amyotrophic lateral sclerosis (Raheja et al., 2018). Previously, significant association of miR-320 gene locus was determined in a genome-wide linkage analysis in patients with late-onset familial AD (Kunkle et al., 2016).

In mouse models of AD, increased expression of miR-320 in brain neurons was determined (Boese et al., 2016). Reduced level of miR-4487 (derived from L1 (Wei et al., 2016)) was detected in neurons of the brain of AD patients (Hu et al., 2018). In AD, miR-384 (derived from LINE/Dong-R4 (Wei et al., 2016)) is overexpressed. This microRNA interacts with mRNA of BACE1 protein (beta-secretase, which catalyzes the conversion of amyloid precursor to beta-amyloid (Samadian et al., 2021)). In the blood serum of AD patients, a reduced level of miR-4286 (Henriques et al., 2020), derived from ERVL (Wei et al., 2016), and miR-4422-5p (derived from LTR/ Gypsy (Wei et al., 2016)) was detected (Hajjari et al., 2021).

In 2019, in the search for potential biomarkers and therapeutic agents for AD, integration of transcriptomic data with protein-protein and transcriptional regulatory interactions revealed the role of miR-192-5p (derived from L2) and miR-335-5p (derived from SINE/MIR) (Wei et al., 2016) as key signaling and regulatory molecules associated with transcriptional changes in AD. Their levels decrease both in the blood of people with AD (Rahman et al., 2019) and in relation to miR-192-5p in the hippocampus of experimental mice. Further studies showed the potential protective efficacy of miR-192-5p in AD. The level of this microRNA decreased with exercise and contributed to a decrease in the expression of TNF-α, IL-6 and IL-1β, which are involved in inflammation in AD (Qin et al., 2022). Similar results were obtained in experiments on cell cultures and AD mouse models regarding miR-335-5p, which can be used for targeted therapy of the disease (Wang et al., 2020).

In 2020, reduced expression of miR-340 (derived from TcMar-Mariner DNA TE (Wei et al., 2016)) was detected in mouse AD models (Tan et al., 2020). A low level of miR- 708-5p (derived from L2 (Wei et al., 2016)) was detected in blood samples of 28 patients with AD (Rahman et al., 2020). The data obtained were confirmed by studying the brain neurons of AD patients (Di Palo et al., 2022). Analysis of brain samples from patients who died from AD showed increased levels of miR-1202 (Henriques et al., 2020), derived from L1 (Wei et al., 2016).

In 2021, an analysis of DNA blood samples from 48 AD patients and 48 healthy controls showed a significant increase in the level of miR-378a (Dong Z. et al., 2021), which was derived from SINE/MIR (Wei et al., 2016). This microRNA has been proposed as a biomarker for AD. The brains of deceased AD patients showed reduced levels of miR-1271 (Majumder et al., 2021), which was derived from L2 (Wei et al., 2016). An increase in the expression of miR-4504 (derived from L1) in the brain of patients with AD was determined (Eysert et al., 2021). Our data on changes in the expression of specific microRNAs derived from TEs in AD are shown in Table 1.

**Table 1.**
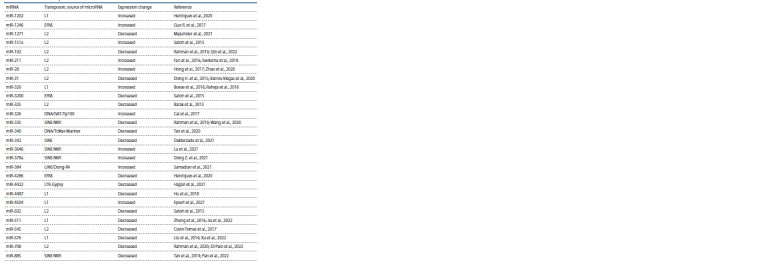
Association of transposon-derived microRNAs with Alzheimer’s disease

## Association of transposable element-derived microRNAs with aging, carcinogenesis and Alzheimer disease

Epidemiological studies indicate a significant increase in the risk of developing AD with age (Niu et al., 2017). In both aging and neurodegenerative diseases, genomic instability is observed in neurons, with activation of TEs by various mechanisms (Wood, Helfand, 2013; Guo C. et al., 2018), including loss of SIRT6 marks (Van Meter et al., 2014). Although AD and cancer are diseases associated with aging, an analysis of scientific literature has identified an inverse correlation between cancer and AD, which may be due to the influence of the proteins p53 and PIN1 (Peptidyl-prolyl cis-trans isomerase) (Lanni et al., 2021). At the same time, mortality from AD in people who survived cancer for 10 years or more was higher than in the general population (Abdel-Rahman, 2020), which may indicate the presence of common pathogenetic pathways of these diseases, possibly associated with TEs deregulation.

Changes in TEs activity during aging contribute to changes in the expression of microRNAs, which can contribute to the development of AD and suppress the growth of cancer (acting as tumor suppressors). To test this assumption, we analyzed an online resource created in 2018 by N.W. Wong et al. concerning changes in specific microRNAs in certain cancer types (Wong et al., 2018), as well as searched for scientific data on microRNAs associated with AD and aging. As a result, we identified 13 specific microRNAs derived from TEs associated with aging and simultaneously involved in the pathogenesis of AD and cancer (Table 2).

**Table 2.**
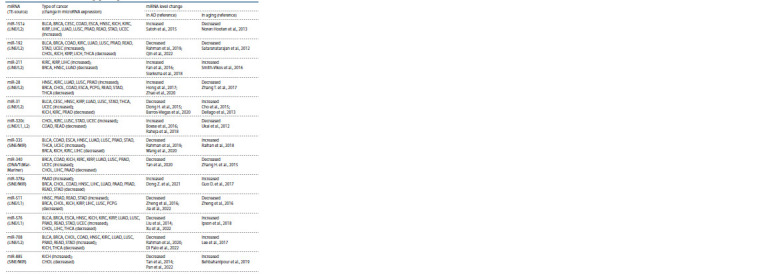
TE-derived miRNAs associated with aging, carcinogenesis and Alzheimer’s disease Notе. BLCA – bladder urothelial carcinoma; BRCA – breast invasive carcinoma; CESC – cervical squamous cell carcinoma and endocervical adenocarcinoma; CHOL – cholangiocarcinoma; COAD – colon adenocarcinoma; ESCA – esophageal carcinoma; HNSC – head and neck squamous cell carcinoma; KICH – kidney chromophobe carcinoma; KIRC – kidney renal clear cell carcinoma; KIRP – kidney renal palillary cell carcinoma; LIHC – liver hepatocellular carcinoma; LUAD – lung adenocarcinoma; LUSC – lung squamous cell carcinoma; PAAD – pancreatic adenocarcinoma; PRAD – prostate adenocarcinoma; PCPG – pheochromocytoma and paraganglioma; READ – rectal adenocarcinoma; STAD – stomach adenocarcinoma; THCA – thyroid carcinoma; UCEC – uterine corpus endometrial carcinoma.

MiR-151a is associated with Alzheimer’s disease (Satoh et al., 2015); expression changes of this microRNA are also characteristic of various cancers (Wong et al., 2018) and aging (Noren Hooten et al., 2013). LINE2-derived miR-192 (Wei et al., 2016), the level of which decreases in AD (Rahman et al., 2019; Qin et al., 2022), is associated with various cancers (Wong et al., 2018). miR-192 expression is significantly reduced in aging kidney tissue (Sataranatarajan et al., 2012). LINE/L2-derived miR-211 (Wei et al., 2016), the level of which increases in AD (Fan et al., 2016; Sierksma et al., 2018), is also associated with cancer (Wong et al., 2018). miR-211 expression is increased in centenarians and may serve as a biomarker of aging (Smith-Vikos et al., 2016).

LINE2-derived miR-28 (Wei et al., 2016), the level of which increases in AD (Hong et al., 2017; Zhao et al., 2020), is also associated with specific cancers (Wong et al., 2018). Physiological aging is associated with decreased miR-28 production (Zhang T. et al., 2017). LINE2-derived miR-31 (Wei et al., 2016), the level of which decreases in AD (Dong H. et al., 2015; Barros-Viegas et al., 2020), is associated with cancer (Wong et al., 2018). Expression of this microRNA is increased during replicative aging (Dellago et al., 2013). MiR-320c, derived from LINE2 (Wei et al., 2016), the level of which is increased in AD (Boese et al., 2016; Raheja et al., 2018), is also associated with specific cancers (Wong et al., 2018). MiR-320c levels decrease with aging (Ukai et al., 2012).

MiR-335, derived from SINE/MIR (Wei et al., 2016), which is reduced in AD (Rahman et al., 2019; Wang et al., 2020), is also associated with cancer (Wong et al., 2018) and aging (Raihan et al., 2018). MiR-340, derived from DNA-TE TcMar- Mariner (Wei et al., 2016), the expression of which is reduced in AD, is associated with cancer (Wong et al., 2018) and with aging (Zhang H. et al., 2015). SINE/MIR-derived miR-378a (Wei et al., 2016), the level of which is significantly increased in AD (Dong Z. et al., 2021), is associated with various cancers (Wong et al., 2018) and aging (Guo D. et al., 2017). The level of miR-511 (source: L1 (Wei et al., 2016)) decreases not only in AD (Zheng et al., 2016; Jia et al., 2022), but also in aging. This microRNA is also associated with cancer (Wong et al., 2018). L1-derived miR-576 (Wei et al., 2016), the level of which is reduced in AD (Liu et al., 2014; Xu et al., 2022), is associated with cancer (Wong et al., 2018).

Increased expression of miR-576 is detected during aging (Ipson et al., 2018). MiR-708, derived from LINE2 (Wei et al., 2016), a reduced level of which is observed in AD (Rahman et al., 2020; Di Palo et al., 2022), is associated with specific cancers (Wong et al., 2018) and with aging (Lee et al., 2017). The SINE/MIR-derived microRNA miR-885, which is downregulated in AD (Tan et al., 2014), is associated with chromophobe kidney cancer and cholangiocarcinoma (Wong et al., 2018) and aging (Behbahanipour et al., 2019).

## Conclusion

The role of microRNAs in the development of AD indicates the possible potential of targeted therapy for the disease, as well as the search for the most optimal treatment regimens for AD. Examples are the decrease in miR-192-5p levels during exercise, which contributes to suppression of pro-inflammatory cytokines TNF-α, IL-6 and IL-1β production (Qin et al., 2022); the decrease in miR-28-3p levels after donepizil therapy (which can be used as a diagnostic criterion for the effectiveness of treatment) (Zhao et al., 2020).

MicroRNAs can become not only therapeutic agents, but also highly accurate diagnostic markers, since changes in their levels are accompanied by regression in AD clinical picture, as was shown for miR-511 when exposed to acupuncture moxibustion in AD (Jia et al., 2022). The experimental effectiveness of miR-31 (Barros-Viegas et al., 2020) and miR-335-5p (Wang et al., 2020) in significantly reducing beta-amyloid accumulation in the hippocampus and its base indicates the potential of using this microRNA for targeting AD therapy (Barros-Viegas et al., 2020; Wang et al., 2020).

The association of TE-derived miRNAs with Alzheimer’s disease indicates both the promise of their use in the treatment of AD and the need for a more detailed study of the mechanisms of action of these miRNAs, since the complementarity of their sequences with various TEs may become a likely basis for global changes in the expression of genes under TEs regulatory control.

## Conflict of interest

The authors declare no conflict of interest.
